# Transcriptome of the adult female malaria mosquito vector *Anopheles albimanus*

**DOI:** 10.1186/1471-2164-13-207

**Published:** 2012-05-30

**Authors:** Jesús Martínez-Barnetche, Rosa E Gómez-Barreto, Marbella Ovilla-Muñoz, Juan Téllez-Sosa, David E García López, Rhoel R Dinglasan, Ceereena Ubaida Mohien, Robert M MacCallum, Seth N Redmond, John G Gibbons, Antonis Rokas, Carlos A Machado, Febe E Cazares-Raga, Lilia González-Cerón, Salvador Hernández-Martínez, Mario H Rodríguez López

**Affiliations:** 1 Centro de Investigación sobre Enfermedades Infecciosas, Instituto Nacional de Salud Pública, Cuernavaca, Morelos, México; 2 Johns Hopkins Bloomberg School of Public Health. Department of Molecular Microbiology & Immunology, Johns Hopkins Malaria Research Institute, Baltimore, MD, 21205, USA; 3 Department of Molecular & Comparative Pathobiology, Johns Hopkins University School of Medicine, Baltimore, MD, USA; 4 Division of Cell and Molecular Biology, Department of Life Sciences, Imperial College London, London, United Kingdom; 5 Pasteur Institut, 28 Rue Du Docteur Roux, Paris, 75015, France; 6 Department of Biological Sciences, Vanderbilt University, Nashville, TN, USA; 7 Department of Biology, University of Maryland, College Park, MD, USA; 8 Departamento de Infectómica y Patogénesis Molecular, Cinvestav-IPN, México, DF, México; 9 Centro Regional de Investigación en Salud Pública, Instituto Nacional de Salud Pública, Tapachula, Chiapas, México

**Keywords:** *Anopheles albimanus*, Transcriptome, Malaria, RNA-Seq

## Abstract

**Background:**

Human Malaria is transmitted by mosquitoes of the genus *Anopheles*. Transmission is a complex phenomenon involving biological and environmental factors of humans, parasites and mosquitoes. Among more than 500 anopheline species, only a few species from different branches of the mosquito evolutionary tree transmit malaria, suggesting that their vectorial capacity has evolved independently. *Anopheles albimanus* (subgenus *Nyssorhynchus*) is an important malaria vector in the Americas. The divergence time between *Anopheles gambiae*, the main malaria vector in Africa, and the Neotropical vectors has been estimated to be 100 My. To better understand the biological basis of malaria transmission and to develop novel and effective means of vector control, there is a need to explore the mosquito biology beyond the *An. gambiae* complex.

**Results:**

We sequenced the transcriptome of the *An. albimanus* adult female. By combining Sanger, 454 and Illumina sequences from cDNA libraries derived from the midgut, cuticular fat body, dorsal vessel, salivary gland and whole body, we generated a single, high-quality assembly containing 16,669 transcripts, 92% of which mapped to the *An. darlingi* genome and covered 90% of the core eukaryotic genome. Bidirectional comparisons between the *An. gambiae*, *An. darlingi* and *An. albimanus* predicted proteomes allowed the identification of 3,772 putative orthologs. More than half of the transcripts had a match to proteins in other insect vectors and had an InterPro annotation. We identified several protein families that may be relevant to the study of *Plasmodium*-mosquito interaction. An open source transcript annotation browser called GDAV (Genome-Delinked Annotation Viewer) was developed to facilitate public access to the data generated by this and future transcriptome projects.

**Conclusions:**

We have explored the adult female transcriptome of one important New World malaria vector, *An. albimanus.* We identified protein-coding transcripts involved in biological processes that may be relevant to the *Plasmodium* lifecycle and can serve as the starting point for searching targets for novel control strategies. Our data increase the available genomic information regarding *An. albimanus* several hundred-fold, and will facilitate molecular research in medical entomology, evolutionary biology, genomics and proteomics of anopheline mosquito vectors. The data reported in this manuscript is accessible to the community via the VectorBase website (http://www.vectorbase.org/Other/AdditionalOrganisms/).

## Background

Human malaria transmission is dependent on efficient development of *Plasmodium* parasites within anopheline mosquito vectors. Anopheline mosquitoes are a large subfamily comprising nearly five hundred species distributed in subtropical and tropical areas around the world, but only a small percentage (10-20%) are malaria vectors [1]. Intriguingly, malaria infection rates among anopheline species do not correlate with mosquito phylogenetic relationships, suggesting that genetic traits associated with vectorial capacity have quickly and independently evolved in different species [2].

Vectorial capacity is a highly complex biological phenomenon depending on mosquito behavior, lifespan and innate refractoriness or susceptibility to *Plasmodium* infection, which may result from the co-evolutionary forces driving the tripartite interaction between humans, mosquitoes and parasites [3]. As a result of the sequencing of the *Anopheles gambiae* and *Plasmodium falciparum* genomes, a great deal of information regarding our understanding of mosquito-pathogen interactions at a molecular level has been gained [4,5]. Post genome research has highlighted the role of the *An. gambiae* innate immune response in determining mosquito refractoriness to *Plasmodium*[6-8].

In addition to the mosquito’s innate ability to transmit malarial parasites, critical aspects of mosquito biology, like adaptation to diverse niches, host seeking behavior, and resistance to insecticides are still unknown. Novel strategies for control, based on a deep understanding of mosquito biology and evolution, will be required to achieve the goal of eventual malaria eradication. Rapid technological advances in DNA sequencing, protein characterization by mass spectrometry and bioinformatics offer unique opportunities to generate large catalogs of genes, proteins and biological networks that may enable the identification of potential mosquito control targets beyond the *An. gambiae-P. falciparum* dyad [9], which can be harnessed by novel strategies such as transgenesis, mosquito-based transmission blocking vaccines [10,11] and alternative insecticides [12].

*Plasmodium vivax* malaria still represents a major health and socio-economic burden in Asia, the Western Pacific and the Americas [13,14]. Malaria in the Americas is transmitted by several anopheline species, including species belonging to the *Nyssorhynchus* subgenus, which is unique to the New World. *Anopheles (Nyssorhynchus) albimanus* is a major vector in southern México, Central America and the northern region of South America [15,16]. The *Nyssorhynchus* subgenus is thought to be the earliest diverged branch of the anopheline radiation, which probably occurred more than one hundred million years (My) ago when the supercontinent Gondwana separated to give rise to the actual South American and African continents [17]. The proposed independent emergence of vectorial traits in conjunction with their rapid evolution may imply different molecular strategies involved in *P. vivax* and *P. falciparum* refractoriness and susceptibility in this subgenus. However, very little molecular information exists for any of the New World anopheline vectors, except for *Anopheles (Nyssorhynchus) darlingi* whose genome draft was recently released [18]. The *An. albimanus* genome is scheduled for genome sequencing in the near future [2].

We describe herein the results of a gene discovery based cDNA sequencing project combining conventional Sanger with Next Generation Sequencing (NGS) platforms to analyze cDNA samples derived from *An. albimanus* tissues including the midgut, cuticular fat body, dorsal vessel and salivary gland, which are critical organs involved in the *Plasmodium* life cycle [6,19,20] (Hernández-Martínez, *Unpublished observations*). The main objective of our study was to construct a reference transcriptome that will facilitate molecular and applied studies of *An. albimanus* refractoriness to *Plasmodium* infection and other biological processes relevant for disease transmission. Finally, to maximize accessibility for this and future transcriptome sequencing projects in the absence of a genome sequence, we developed an open-source sequence annotation browser called GDAV (Genome-Delinked Annotation Viewer; http://funcgen.vectorbase.org/gdav). The *An. albimanus* transcriptome annotations are available via the VectorBase website (http://www.vectorbase.org/Other/AdditionalOrganisms/).

## Results and discussion

### The *An. albimanus* transcriptome assembly

**Figure 1:**
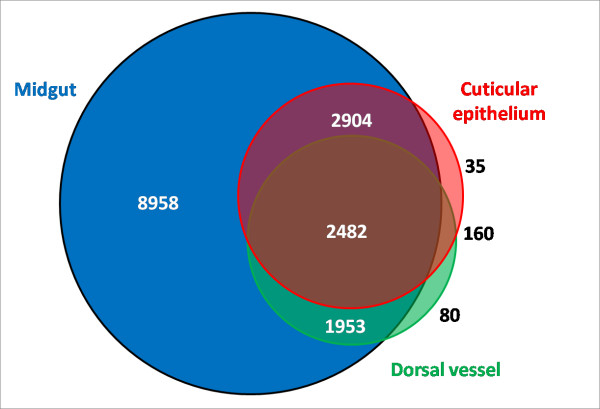
**Origin of transcripts according to mosquito organ.** Distribution of transcript number (n = 16,572) according to the mosquito organ. The area of each circle is roughly a proportional approximation of the number of transcripts. A marked bias towards midgut is noted due to the enormous throughput of Illumina sequencing of a midgut library. Libraries sequenced by Sanger were left out given the low throughput of Sanger sequencing and for simplicity. Thus, not all the 16,669 transcripts are represented.

To capture as much of the transcriptome of the tissues involved in the interaction with *Plasmodium spp.* as possible, we combined transcriptome data generated with Sanger, 454 and Illumina sequencing platforms from several different sources of adult female *An. albimanus* RNA. Table 1A describes the origin of the RNA used to generate cDNA libraries sequenced by the different platforms, as well as the read number contribution of each. Owing to the inherent differences in throughput of each of the sequencing platforms used, most of the final dataset contains Illumina reads derived from the midgut transcriptome (94%), and nearly half of the transcripts were built with at least one 454 read. Only 6% of the built transcripts contained one or more Sanger reads (Table 1B). There were 8,958 (54%) transcripts expressed exclusively in the midgut, 35 (0.2%) were derived specifically from cuticular epithelium/fat body, and 80 (0.47%) were expressed only in the dorsal vessel, whereas the rest (45%) were found in two or more tissues (Figure 1).

**Table 1:** Libraries, sequencing metrics and assembly

**A: Tissue and sequencing platform**			
**Tissue source**	**Illumina (reads)**	**454**	**Sanger**
		**(reads)**	**(reads)**
**Midgut**	210 x10^6^	62,703^**c,e**^	1,017
**Abdominal cuticle**^**a**^		111,566^**c**^	605
**Dorsal vessel**		233,984^**d**^	
**Salivary gland**			1,038
**Whole female**^**b**^			2,121
**Totals**	**210 x10**^**6**^	**408,253**	**4,781**
**B: Transcript composition**			
		**Transcripts**	**(%)**
**Transcripts with Illumina reads**		15,764	94.4
**Transcripts with 454 reads**		8,060	48.3
**Transcripts with Sanger reads**		1,051	6.3
**C: Transcript assembly summary**			
**Total transcripts**			16,699
**Total bases (Mbp)**			16.1
**Mean contig length (bp)**			970
**N50 (bp)**			1,434

^**a**^ Includes abdominal fat body.

^**b**^ Inoculated with *S. marcescens.*

^**c**^ Flx. 250 bp read length.

^**d**^ Titanium 400 bp read length.

^**e**^*P. vivax* infected and non-infected.

An initial transcriptome assembly was performed by combining all sequence reads described in Table 1A, which generated 15,764 contigs. Re-mapping the Sanger and 454 reads to the initial assembly revealed that a substantial number of 454 (53%) and Sanger reads (44%) were not represented in the initial transcriptome assembly, so they were re-assembled using GS Assembler, generating an additional set of 935 contigs. The total transcript dataset included 16,699 contigs with a mean contig length of 970 bp and a N50 of 1,434 bp (Table 1C). Our assembly metrics closely resemble the recently published NGS and Sanger data on the *An. funestus* transcriptome, which yielded 15,527 contigs with a N50 of 1,753 bp [21]. Similar results were also reported in *de novo* transcriptome assembly studies for the planarian worm, *Schmidtea mediterranea* in which 454 reads were pre-assembled to serve as scaffolds for Illumina paired-end assembly, yielding 17,465 contigs with a N50 of 1.6 kb [22], and 18,619 contigs with a mean length of 1,118 bp [23].

**Figure 2:**
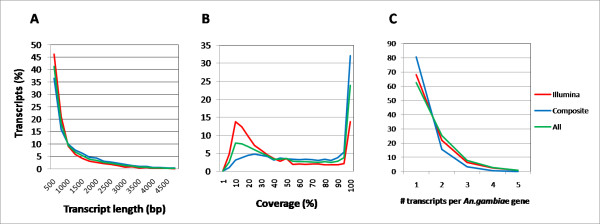
**Transcript length and protein length coverage of the* An. albimanus  An. albimanus *transcriptome.** The whole transcriptome was split in two subsets according to transcripts composed of Illumina reads only or Illumina plus either of the two other platforms (composite subset). To make datasets comparable, their frequency (*y* axis) was expressed as a percentage. **A)** Transcript length distribution for both subsets and the whole dataset reveals slightly longer transcripts in the composite subset. **B)***An. gambiae* protein length coverage obtained by BLASTX with *An. albimanus* transcripts. A larger fraction of the Illumina only subset covers less than one third of its respective match in *An. gambiae*. **C)** Frequency of mapped *An. albimanus* transcripts per *An. gambiae* gene. Transcripts were mapped to the *An. gambiae* genome using Exonerate v 2.2 [79] in the EST to genome mode (DNA vs DNA).

As a surrogate approach to estimate the contribution of each sequencing platform to the assembly quality, the transcript dataset was split into two subsets according to whether transcripts were assembled with only Illumina reads or with Illumina and 454 or Sanger reads. The Illumina-only subset was composed of 8,245 transcripts with a mean contig length of 822 bp and a N50 of 1,111 bp. The composite subset (8,445 transcripts build up with Illumina + 454 or Sanger) had a mean contig length of 1,087 bp and a N50 of 1,648 bp (Figure 2A), which is similar to the recently published *Aedes albopictus* transcriptome using 454 sequencing [24]. We then evaluated if there were differences in the proportion of homolog proteins in the *An. gambiae* predicted proteome using BLASTX (e value of 1.0 E^-5^) between both subsets. We did not observe a difference in the proportion of transcript matches between the Illumina-only subset and the composite ones (51% and 54%, respectively). However, the *An. gambiae* protein length coverage of translated transcripts was considerably improved in those transcripts belonging to the composite subset (Figure 2B) since 53% of their transcripts covered more than 70% of the *An. gambiae* target, whereas only 25% of the Illumina-only transcripts had an equivalent *An. gambiae* target coverage.

### Genome mapping results to *An. gambiae* and *An. darlingi*

**Figure 3:**
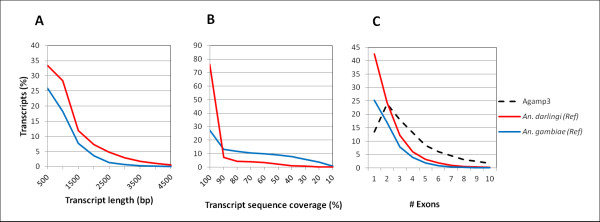
**Reference genome mapping of the *An. albimanus *transcriptome.** Transcripts were mapped to the *An. gambiae* (blue line) or the *An. darlingi* (red line) genomes using Exonerate v 2.2 as in **Figure**2C. Mapped transcripts are expressed as a percentage of the total *An. albimanus* dataset. **A)** Aligned length plot; **B)** Fraction of the *An. albimanus* transcripts that aligned to either genome; **C)** Putative exon number relative to the proportion of *An. albimanus* mapped transcripts. For comparison, distribution of exon frequency in *An. gambiae* is shown (dotted black line).

Since there is no genome sequence available for *An. albimanus*, unambiguous sequence alignment of *An. albimanus* transcripts to a reference anopheline genome could provide additional measures of the transcriptome assembly accuracy and completeness. Moreover, it could provide the means for refining reference genome annotation and provide evidence of functionally conserved genes that were missed by current gene finding algorithms [25]. The final cDNA assembly was mapped to the *An. gambiae* (100 My divergence, subgenus *Cellia*) and *An. darlingi* genomes (closer relative from subgenus *Nyssorynchus*). The current version of the *An. gambiae* PEST strain genome (AgamP3.6) is 278.2 Mbp long and it is in an advanced stage of assembly (oriented scaffolds) [26] and annotation (13,320 predicted genes) [27]. The *An. darlingi* genome was sequenced using 454 sequencing and is in an early stage of assembly (3,990 un-oriented contigs) and annotation (11,430 predicted protein coding genes) [18]. Using transcript to genome DNA alignment (*see methods*), 15,441 *An. albimanus* transcripts (92%) aligned (Exonerate bestn score >300) to the *An. darlingi* genome. Expressed as aligned base pairs, 13.8 of 16.1 Mbp (85%) of the *An. albimanus* transcriptome aligned to the *An. darlingi* genome (Table 2). Transcriptome mapping with the same parameters to the *An. gambiae* genome resulted in 9,648 aligned contigs (58%) with 7.1 Mbp (46%) aligned. Contigs that aligned to *An. darlingi* but not to the *An. gambiae* genome were predominantly short contigs (0.3-1.5 Kb) (Table 2. Figure 3A). As expected from the overall transcriptome aligned fraction, the fraction of *An. albimanus* transcript sequences that aligned to *An. darlingi* (transcript coverage) was considerably higher than for the *An. gambiae* genome, such that 76% of *An. albimanus* transcripts that were mapped to the *An. darlingi* genome aligned with more than 90% of their respective length (Figure 3B). Our mapping results using *An. darlingi* as the reference genome are significantly better than those described for the planarian worm *S. mediterranea*, which were mapped to its own genome [23], suggesting that despite its early stage of annotation, the *An. darlingi* genome is an appropriate surrogate genome for *An. albimanus*, and supports the accuracy and quality of our assembly. Transcripts that did not map to the *An. darlingi* genome (8%), as well as partial alignments may represent mis-assemblies in the transcriptome or the genome, rapidly evolving genes or the rapid evolution of untranslated regions (UTRs). Conversely, poor mapping to the *An. gambiae* reference genome may be result of all the above, further confounded by the increased evolutionary distance separating the two species.

**Table 2:** **Transcript mapping to *Anopheles*genomes**^**a**^

**Reference genome**	***An. gambiae***		***An. darlingi***	
**Unique alignments**	9,648	**58**^**b**^	15,441	**92**
**Aligned bases (Mbp)**	7,3	**46**	13,8	**85**
**Number of transcript with introns**	5,438	**33**	8,365	**50**
**Transcripts mapping to genes**	6,305		ND	
**Single transcript per*An. gambiae*gene**	3,949	**62**^**c**^	ND	
**Single Illumina-only per*An. gambiae*gene**	2,707	**68**^**d**^	ND	
**Single composite per*An. gambiae*gene**	3,386	**80**^**d**^	ND	

^a^ Mapping was done using Exonerate v 2.2. using the EST2genome mode (DNA transcript aligned to genomic DNA)[79].

^b^ Numbers in bold letters indicate the fraction (%) of *An. albimanus* dataset

^c^ Percentage of total mapped.

^d^ Percentage of corresponding subset.

An analysis of the protein length coverage for *An. albimanus-An.gambiae* BLASTX alignments showed a considerable proportion of transcripts with only partial coverage of their corresponding *An. gambiae* match (Figure 2B), indicating the possibility of multiple hits per *An. gambiae* gene. The extent of multiple transcripts mapping to a single *An. gambiae* gene was estimated by comparing transcript to genome and *An. gambiae* gene coordinates. *Anopheles albimanus* transcripts mapped partially or fully within the sequences of 6,305 *An. gambiae* genes, and 62% of these transcripts did so within a single *An. gambiae* gene (Table 2. Figure 2C). Based on our protein length coverage observations (Figure 2B), we noted a higher proportion of single gene hits in the composite subset (80%) than in the Illumina-only subset (68%) (Figure 2C).

As an additional approach to estimate transcript completeness, we used reference genome mapping to analyze exon coverage. As expected from the phylogenetic relationships, transcript mapping to the *An. darlingi* genome showed higher average exon coverage (2.1 exons per transcript) than to the *An. gambiae* genome (1.9 exons per transcript), which falls below the 4.4 exons per transcript in *An. gambiae*[28] (Figure 3C). However, there was no proportional difference in the exon coverage using *An. gambiae* or *An. darlingi* relative to the number of significant alignments (data not shown). More than half (54.1%) of our mapped transcript dataset covers at least two exons, whereas in *An. gambiae*, more than 86% of the genes are composed of at least two exons (Figure 3C). Only ten and thirty-one transcripts that mapped to *An. gambiae* and *An. darlingi*, respectively, had more than 10 exons, compared to 742 for *An. gambiae*. The *An. albimanus* transcript that covered most exons (23) when mapped to the *An. darlingi* genome was Locus_3073_Length_3,997, and corresponds to the *An. gambiae* AGAP000009 homolog, which is predicted to have 25 exons (data not shown).

In summary, transcript mapping to reference genomes and the derived analysis of exon structure of our transcript dataset revealed a degree of incompleteness when using *An. gambiae* as our reference. The increased proportion of genome alignments without a spanning intron (single exon transcripts) observed in *An. albimanus* could result from incorrect splitting of the transcript during the assembly of transcriptional units having more than one exon. Correct exon representation is relevant because alternative splicing is a means to increase proteome diversity and this phenomenon has been observed frequently among dipterans [29,30]. Incomplete exon-exon structure across *An. albimanus* transcripts could underestimate the diversity of protein configurations and thus, may limit protein identification by proteomic approaches in the absence of the genome, and empirical studies to assess this possibility are required to fill in this gap in knowledge. Despite the limitations in our dataset, information regarding exon-exon structure may be useful for experimentalists when designing primers and probes for one gene-targeted analysis.

### Estimated proteome coverage

As a starting point for transcript annotation, the proportion of the *An. albimanus* transcriptome that was homologous to a predicted protein sequence in other genomes was analyzed. Protein similarity to other insect proteomes and the NCBI nr databases was assessed using BLASTX (using an e-value threshold of 1.0E^-5^). A total of 10,000 sequences (62%) in our dataset had a significant match with at least one species. However, we note that probably due to methodological differences, this proportion is lower than the 84% match described by Crawford, when the *An. funestus* transcriptome was compared with the *An. gambiae* proteome [21].

**Figure 4:**
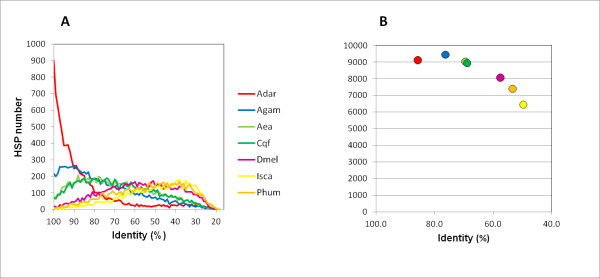
**Proteome comparison to other insect proteomes.** The *An. albimanus* transcriptome was compared to insect proteomes using BLASTX (e val. 1.E^-05^). **A)** Distribution of protein identity (%) of all high scoring pairs (HSP) for *An. darlingi* (Adar, red), *An. gambiae* (Agam, blue), *Ae. aegypti* (Aea, light green), *C. quinquefasciatus* (Cqf, dark green), *D. melanogaster* (Dmel, pink); *I. scapularis* (Isca, yellow) and *P. humanus* (Phum, orange). **B)** Average protein identity of *An. albimanus* best hits to insect proteomes. Color codes for each species as in **A**.

Contrary to what would be expected based on degree of evolutionary relationships, and the results observed in transcript to genome alignments, we observed that a higher proportion of *An. albimanus* transcripts (56%) had a match with *An. gambiae* than with *An. darlingi* (54%) (Table 3. Figure 4B). However, a comparison between *An. darlingi* and *An. gambiae* revealed that 80% of the *An. darlingi* proteins matched the *An. gambiae* proteome. This can be partially explained because the *An. albimanus* predicted proteome is considerably larger (16,699) than the *An. darlingi* predicted proteome.

**Table 3:** BLASTX comparisons to other Insect predicted proteomes

	***An. albimanus*matchs**	**%**	**Average identity %**	**SD**	**Median Identity (%)**	**Reference proteome size**	**Reference Proteome coverage (%)**
***An. darlingi***	9,116	**54.6**	85.8	17.1	92.0	11,764	48.4
***An. gambiae***	9,445	**56.6**	76.3	17.8	80.3	12,670	51.7
***Ae. aegypti***	9,030	**54.1**	69.5	18.4	71.7	15,988	40.3
***C. quinquefasciatus***	8,936	**53.5**	68.8	17.1	70.9	18,883	33.6
***D. melanogaster***	8,069	**48.3**	57.5	17.9	57.0	13,804	41.9
***I. scapularis***	6,446	**38.6**	49.7	16.6	47.4	20,486	21.7
***P. humanus***	7,396	**44.3**	53.4	17.7	51.4	10,783	46.0

Considering the total amount of protein coding genes in each of the sequenced vector genomes, 51% of *An. gambiae* and 48% of the *An. darlingi* protein coding genes matched at least one transcript in *An. albimanus*, whereas *An. darlingi* covered 62% of the *An. gambiae* proteome. Proteome coverage tended to decrease according to phylogenetic distance (Figure 4B). However, the proportion of significant best hits decreased according to phylogenetic relatedness, so that protein percent identity among best BLASTX hits was the highest with *An. darlingi* (average identity 85%), and decreased according to phylogenetic distance (Table 3. Figure 4A-B).

The lower number of transcript matches observed between *An. albimanus* and *An. darlingi* than between *An. albimanus* and *An. gambiae* is likely the consequence of both the incompleteness of the *An. albimanus* dataset due to sampling of only a limited set of tissues from adult mosquitoes, and the current incomplete assembly status of the *An. darlingi* genome (Figure 4B). Moreover, the *An. gambiae* genome annotation relied heavily on homology based annotation approaches [5], and thus is very conservative. This implies that rapidly evolving genes may escape identification by such conservative approaches. Combining conservative and *ab initio* gene prediction strategies increased considerably the amount and quality of the *An. gambiae* protein coding genes [25]. Given the fact that most of the *An. albimanus* transcripts mapped to the *An. darlingi* genome, many transcripts may in fact be derived from true protein coding genes. Additionally, many of the sequences could represent non-protein coding transcripts of potential biological significance [31].

### Orthologs

The proportion of the core eukaryotic genome covered by the *An. albimanus* transcriptome was investigated by searching for the 458 core eukaryotic protein models [32] in the *An. albimanus* predicted proteome, as well as the predicted proteomes of *An. darlingiAn. gambiaeAe. aegyptiC. quinquefasciatus D. melanogaster, I. scapularis, P. humanus and Rhodnius prolixus.* As expected, we observed almost complete coverage for *D. melanogaster* (99%) and *An. gambiae* genomes (98%). We identified 415 core eukaryotic genes (CEGs) in the *An. albimanus* dataset corresponding to 90% coverage. Coverage ranged from 95-98% in the other genome sequenced species. The lowest coverage was observed for *An. darlingi* (88%), which as discussed in the previous section, may reflect the early stage of that sequencing project (Table 4).

**Table 4:** Proportion of the core eukaryotic genome

**Species**	**CEGs**	**(%)**	**CEGs in 1:1:1 BRH dataset**	**(%)**	**CEGs in 1:1 BRH.*An. albimanus***	**(%)**	**CEGs in 1:1 BRH.*An. gambiae***	**(%)**	**CEGs in 1:1 BRH.*An. darlingi***	**(%)**
***An. albimanus***	415	**90.6**	283	**62**	**NA**	**NA**	374	**82**	320	**70**
***An. gambiae***	453	**98.9**	283	**62**	374	**82**	**NA**	**NA**	361	**79**
***An. darlingi***	403	**88.0**	295	**64**	333	**73**	384	**84**	**NA**	**NA**
***Ae. aegypti***	449	**98.0**	ND		ND		ND		ND	
***C. quinquefasciatus***	440	**96.1**	ND		ND		ND		ND	
***D. melanogaster***	456	**99.6**	ND		ND		ND		ND	
***I. scapularis***	440	**96.1**	ND		ND		ND		ND	
***P. humanus***	456	**99.6**	ND		ND		ND		ND	
***R. prolixus***	439	**95.9**	ND		ND		ND		ND	

**Figure 5:**
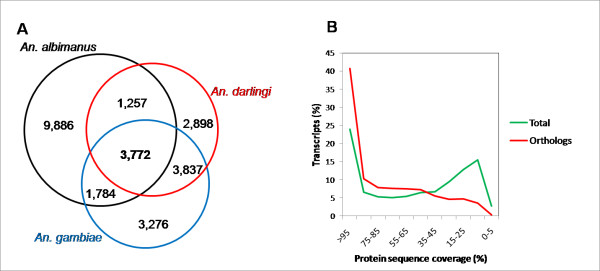
**Ortholog prediction in* An. albimanus*.** Best reciprocal hits were identified by bidirectional BLASTX, BLASTP and TBLASTN comparisons between *An. albimanus* transcriptome, *An. darlingi* and *An. gambiae* genomes. **A)** Venn diagram depicting the numbers of entries for each comparison. The area of each circle is a proportional approximation of the number of transcripts in each. **B)** Protein length coverage frequency distribution in total *An. albimanus* translated transcripts matching the *An. gambiae* proteome (n = 9,445. Green line) and the 1:1:1 putative ortholog dataset (n = 3,772. Red line).

An important goal of the *Anopheles* genome cluster will be to define a “core anopheline genome”. Due to the incompleteness of the predicted proteome for *An. albimanus*, we reasoned that an *An. albimanus - An. gambiae* comparison could be very inaccurate in terms of ortholog prediction. However, using *An. darlingi* as an additional species for bidirectional comparisons, specificity could be increased but at the expense of sensitivity. To identify putative orthologs we used BLASTX and TBLASTN for bidirectional comparisons among the *An. gambiaeAn. darlingi* and *An. albimanus* proteomes. *Anopheles albimanus* - *An. darlingiAn. albimanus* - *An. gambiae* and *An. darlingi* - *An. gambiae* bidirectional comparisons revealed 5,029, 5,556 and 7,609 best reciprocal hits, respectively. The three species comparison yielded a set of 3,772 1:1:1 putative orthologs (32% and 29% of the *An. darlingi* and *An. gambiae* predicted proteome, respectively) (Figure 5A). The 1:1 ortholog dataset between *An. darlingi* and *An. gambiae* comprised 64% and 60% of their proteome, respectively. The proportion of orthologs between *An. gambiae* and *D. melanogaster* is 47 and 44%, respectively [28], and ranged between 73% (*D. melanogaster-D. grimshawi*) to 93% (*D. melanogaster-D. yakuba*) within the *Drosophila* cluster [33].

To further validate the accuracy of our ortholog assignment, we compared protein length coverage, using pair-wise alignments between translated *An. albimanus* proteins and their corresponding best match in *An. gambiae.* A considerable improvement of protein length coverage was observed in orthologs (average protein length coverage was 74%), when compared to the protein coverage of the overall dataset (average protein length coverage of 53%). As described previously (Figure 2B), protein coverage displayed a bimodal distribution where 40% of the translated *An. albimanus* products covered at least two thirds of their corresponding *An. gambiae* matches and another 40% covered less than one third (Figure 5B). A significant and expected improvement was observed in the ortholog dataset, which was biased toward higher coverage, resulting in 66% of the orthologs covering more than two thirds of their corresponding *An. gambiae* matches (Figure 5B).

If our BLAST best reciprocal hit ortholog prediction strategy was accurate, we would expect that all the core eukaryotic genes (CEGs) found would be represented in the 1:1:1 ortholog dataset. As shown in Table 4, only 283 of the identified *An. albimanus* CEGs were included in the three species ortholog dataset, which corresponds to 61% of the core eukaryotic genome. However, the proportion of CEGs found between two species best reciprocal hits was higher and ranged from 70 to 83%. Together, our data indicate that although there is a relatively high coverage of the core eukaryotic genome, the anopheline ortholog assignment remains incomplete (i.e., it is missing a considerable amount of true orthologs), and may be inaccurate (i.e., includes a significant proportion of paralogs).

### Functional annotation of the *An. albimanus* transcriptome

To gain insight into the predicted functional characteristics of the current dataset, translated products were functionally annotated using the Gene Ontology (GO) classification as implemented in the Blast2GO software [34], as well as using the InterPro classification [35]. A total of 5,086 transcripts were annotated according to biological process, and 5,792 transcripts were annotated according to molecular function, which corresponds roughly to one third of all the transcripts in the dataset for both categories, and to one half of the total BLASTX matches in the vectors’ predicted proteomes (Table 3).

**Figure 6:**
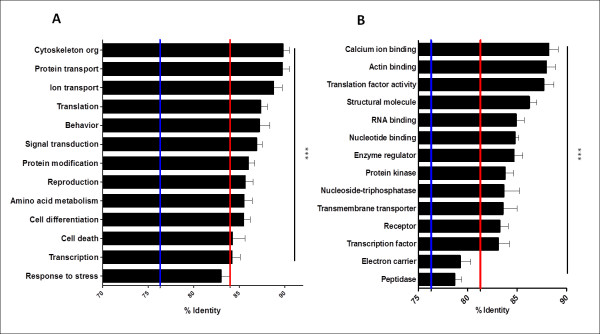
**Protein identity according to Gene Ontology.***An. albimanus* transcriptome was GO annotated using Blast2GO and partitioned according to biological process or molecular function. **A)** Average percent identity of biological function classes with respect to *An. gambiae* in the ortholog dataset. Average protein identity of response to stress and transcription genes was significantly lower than cytoskeleton organization and protein transport genes (*** *P* < 0.001. Kruskal-Wallis test and Dunn’s multiple comparison test). **B)** Average protein identity of best hit matches with respect to *An. gambiae* categorized according to molecular function. Average identity of electron carrier function and Peptidases were significantly lower than calcium and actin binding, translation factor and structural function genes (*** *P* < 0.001. Kruskal-Wallis test and Dunn’s multiple comparison test). For **A** and **B**, red lines indicate the average protein identity of the corresponding GO annotated subset. Blue lines indicate overall protein identity.

To gain insight into the protein evolution rate according to biological process and molecular function, the annotated transcriptome dataset was partitioned according to the most abundant biological process and molecular function GO slim categories. Based on BLASTX matches, average protein percent identity was estimated for each GO slim category, either in the 1:1:1 ortholog dataset, or as in all best hits (homologs) against the *An. gambiae* predicted proteome dataset (data not shown). For both the homologous and orthologous subsets, most conserved proteins belonged to general biological processes such as cytoskeletal organization, ion transport, translation and protein transport (Figure 6A), in agreement with other comparative studies in mosquitoes and *D. melanogaster*[28,36]. Putative homologous and orthologous proteins assigned as having stress response and transcription functions were the least conserved (*P* < 0.05), consistent with the notion of higher evolutionary rates in genes involved in immune response [28,33,37,38], which are a subset of stress response genes. Although the average protein identity observed in the response to stress genes category was lower than the average protein identity in the entire annotated ortholog dataset, such differences were not statistically significant (*P* >0.05). Also, the average protein identity observed in the response to stress genes category was significantly higher relative to the average protein identity in the entire transcriptome (matching proteins in *An. gambiae*) (*P* < 0.5). This may be the result of the high proportion of genes present in our dataset that have no GO annotation and that, not surprisingly, are the least conserved (Figure 6A).

According to molecular function, the best protein matches, those that were involved in translation, structural functions, calcium and actin binding were the most conserved, which agrees with the initial biological process categorization. The least conserved putative proteins were categorized as electron carrier and proteases, whose conservation was significantly lower than that observed for the translation, actin binding and calcium binding (*P* < 0.05) ontologies. Proteases were significantly less conserved than the whole set of annotated predicted proteins (Figure 6B). Proteases have been reported as being subjected to positive selection in the *Drosophila* genus [36]. Also, trypsin family proteases have been significantly expanded in *An. gambiae* compared to *D. melanogaster*, suggesting faster evolution in anophelines that may be related to hematophagy [28,39]. Similarly, electron carrier activity is related to cytochrome function and is overrepresented in our dataset. The cytochrome P450 family, involved in insecticide resistance, is also expanded in *An. gambiae*[40] and may be also evolving rapidly in New World anophelines.

The transcript dataset was also annotated using InterProScan, which yielded 17,850 InterPro annotations. We noted that 7,154 (42%) transcripts have at least one InterPro annotation with an average of 2.4 annotations per annotated transcript. Annotation distribution was similar to *An. gambiae* (Table 5). The most abundant annotations, such as zinc-fingers (IPR007087), WD-40 repeat (IPR001680), Protein kinase domain (IPR011009), Armadillo-like fold (IPR016024) and Serine/Cysteine proteases (IPR009003) and the general substrate transporters of the Major facilitator superfamily (IPR016196), were among the top most frequent annotations for both the *An. albimanus* transcriptome and the *An. gambiae* predicted proteome [41]. This similarity suggests that in general terms and despite being derived from a limited set of adult tissues, the *An. albimanus* dataset exhibits a similar representation to that of *An. gambiae*. However, certain InterPro annotations were more abundant in the *An. albimanus* dataset than in *An. gambiae*. For example, the C-terminal-like Glutathione S-transferase (GST) (IPR010987) was ranked in the 26th place versus 79th in *An. gambiae* and contained almost the complete set (32 out of 38 in *An. gambiae*). Other highly ranked annotations that contained near-complete expected sets were E2 Ubiquitin-conjugating enzymes (IPR000608) (24 of 26); and Peptidase M1, membrane alanine aminopeptidase, (IPR014782) (20 out of 25 in *An. gambiae*). Finally, 41 *An. albimanus* transcripts were annotated as Protein of Unknown Function DUF227 (IPR004119), which ranked among the top 20 most abundant annotated transcripts, whereas in *An. gambiae* this classification was ranked in the 69th place with 43 annotated proteins. Considering the over-representation of midgut-derived transcripts in our dataset (Figure 1), aminopeptidase enrichment is expected due to their proteolytic role in blood digestion [42]. GSTs and other detoxification-related gene expression is particularly enriched in the anterior midgut of *An. gambiae* larvae [43], suggesting that detoxification may also be maintained in the adult midgut. E2 ubiquitin conjugating enzymes are part of a general housekeeping homeostatic mechanism [44], and to our knowledge, they do not play a particular role in midgut physiology. However, in *D. melanogaster*, up- regulation of the ubiquitin-conjugating enzyme homolog coded by *vihar* and the 26 S proteosomal subunit RPN9 in response to dietary Bowman–Birk inhibitor (BBI) intoxication has been described [45]. It can be speculated that overrepresentation of E2 ubiquitin conjugating enzymes in the mosquito midgut may be part of a general stress response to different xenobiotics.

**Table 5:** **Top 50 InterPro Annotations in the*An. albimanus*transcriptome**

**Rank in*An. albimanus***	**Transcripts**	**Description**	**InterPro ID**	**Total hits**	**Rank in*An. gambiae***
1	233	Zinc finger, C2H2-type	IPR007087	2001	1
2	194	Protein kinase-like domain	IPR011009	204	3
3	158	Armadillo-type fold	IPR016024	182	4
4	127	WD40 repeat	IPR001680	605	5
5	106	Serine/cysteine peptidase, trypsin-like	IPR009003	114	2
6	98	Major facilitator superfamily transporter	IPR016196	102	10
7	90	RNA recognition motif, RNP-1	IPR000504	327	12
8	73	Cytochrome P450	IPR001128	361	16
9	68	Thioredoxin-like fold	IPR012336	84	24
10	57	Ankyrin repeat	IPR002110	452	19
11	57	ATPase, AAA + type, core	IPR003593	65	8
12	51	Glycoside hydrolase, catalytic core	IPR017853	52	28
13	48	EF-HAND 2	IPR018249	113	37
14	47	Ras	IPR013753	48	26
15	46	Src homology-3 domain	IPR001452	194	40
16	43	Pleckstrin homology	IPR001849	98	35
17	41	Short-chain dehydrogenase/reductase SDR	IPR002198	133	45
18	41	Protein of unknown function DUF227	IPR004119	46	69
19	40	DEAD-like helicase, N-terminal	IPR014001	40	20
20	38	Ras GTPase	IPR001806	150	26
21	38	Leucine-rich repeat	IPR001611	93	6
22	38	Small GTPase, Rho type	IPR003578	45	48
23	37	Immunoglobulin E-set	IPR014756	48	30
24	36	Tetratricopeptide repeat	IPR019734	275	31
25	36	Immunoglobulin-like	IPR007110	58	13
26	32	Glutathione S-transferase, C-terminal-like	IPR010987	56	79
27	31	PDZ/DHR/GLGF	IPR001478	122	32
28	31	BTB/POZ fold	IPR011333	61	23
29	28	Heat shock protein DnaJ, N-terminal	IPR001623	137	87
30	28	Concanavalin A-like lectin/glucanase	IPR008985	35	43
31	28	Homeodomain-like	IPR009057	35	14
32	27	General substrate transporter	IPR005828	27	49
33	25	Glucose/ribitol dehydrogenase	IPR002347	116	51
34	25	Cellular retinaldehyde-binding, C-terminal	IPR001251	96	53
35	24	Ubiquitin-conjugating enzyme, E2	IPR000608	85	137
36	23	Fibronectin type III domain	IPR008957	66	38
37	23	Histone-fold	IPR009072	44	47
38	22	Chitin binding protein, peritrophin-A	IPR002557	148	22
39	21	Immunoglobulin I-set	IPR013098	38	17
40	20	Peptidase M1, membrane alanine aminopeptidase, N-terminal	IPR014782	67	133
41	19	Fibrinogen, alpha/beta/gamma chain, C-terminal globular	IPR002181	72	57
42	19	GPCR, rhodopsin-like superfamily	IPR017452	19	25
43	17	SH2 motif	IPR000980	108	97
44	17	Carboxylesterase, type B	IPR002018	32	59
45	17	7TM GPCR, rhodopsin-like	IPR000276	77	21
46	16	WW/Rsp5/WWP	IPR001202	100	145
47	16	Protease inhibitor I4, serpin	IPR000215	63	117
48	15	C-type lectin fold	IPR016187	17	75
49	15	Protein-tyrosine phosphatase, receptor/non-receptor type	IPR000242	92	NP^a^
50	14	Peptidase M14, carboxypeptidase A	IPR000834	48	154

^a^ Not present.

Structurally, the DUF227 domain overlaps with the SCOP Protein kinase-like (PK-like) superfamily (IPR011009). The presence of DUF227 containing predicted homologs was investigated by querying electronically inferred orthology in the BioMart database [46] and found that DUF227 is present in a family of proteins well represented in dipterans, nematodes, and to a lesser extent in other insects, but absent in other invertebrates such as the sea urchin. The number of DUF227 containing orthologs according to BioMart decreased with phylogenetic distance so that between *An. gambiae* and *Ae. aegypti* there were sixteen 1:1 orthologs and between *An. gambiae* and *D. melanogaster* or *Pediculus humanus* there were only nine and five, respectively. Using VectorBase and MozAtlas for microarray data mining to identify significant changes in gene expression (*P* < 1.E^-5^) for those *An. gambiae* genes containing the DUF227 domain, we found predominant expression in the Malpighian tubules, midgut and head of adult mosquitoes, with expression levels higher in males than in females [47]. In the midgut, 34 out of 43 genes had a significantly modified expression pattern after a blood meal [42]. Five genes were enriched in hemocytes and another five were enriched in the carcass [48]. Two genes were induced during *P. berghei* midgut invasion [49].

Conversely, certain annotations that are abundantly represented in *An. gambiae* such as Insect cuticle proteins (IPR000618); Tropomyosin (IPR000533); 7TM chemoreceptor (IPR013604); Mitochondrial Rho-like (IPR013684); Olfactory receptor, *Drosophila* (IPR004117); Pollen allergen Poa pIX/Phl pVI, C-terminal (IPR001778) were substantially scarce in our dataset (Table 5). As expected, olfactory receptors, 7TM chemoreceptors, and pollen allergen were underrepresented protein annotations since they are not expressed in the midgut [47]. Similarly, insect cuticle proteins that are involved in adult cuticle synthesis show a peak during metamorphosis and are under-expressed in adults [50].

### Immunity related genes

Mosquito immune responses are thought to play an important role in influencing vectorial competence. Higher protein divergence in genes implicated in stress response (Figure 6) is in agreement with the observation of higher divergence rate in immunity related genes (IRGs) than in genes involved in other core cellular processes [33,36-38]. Thus, we searched for potential immunity related genes by comparing our transcript sequences to a manually curated dipteran IRG dataset, which includes 385 genes classified into 27 families implicated in recognition, regulation, signal transduction and effector phases of the immune response [38,51]. We found 413 best BLASTX reciprocal matches between our dataset and the ImmunoDB, representing all 27 families. However, this number may be an overestimation of the true number of IRGs in our dataset, because it includes a large number of structurally homologous proteins that may not be involved in insect immune responses (for example many proteases and tyrosine kinases). To refine our IRG search, we considered a rather conservative approach that contemplated the putative orthology as described in the previous section (Figure 5A), as well as structural signatures derived from the InterPro annotation. Among our 3,772 ortholog 1:1:1 dataset, 79 ortholog groups have matches in the ImmunoDB (Additional file 1) and 73 were consistently supported by InterPro annotation or other protein family classification system. This set was further increased by the inclusion of three unannotated genes of the Toll and Imd pathways (*TOLLPATH1IMDPATH5* and *IMDPATH8*) with supporting structural evidence based on InterPro annotation. This 82 IRG putative ortholog dataset exhibited 75% average identity to their corresponding *An. gambiae* proteins, which is very similar to the average identity of all *An. albimanus*: *An. gambiae* matches. However, average identity was lower than the average identity of GOslim annotated ortholog proteins (84%).

Gene duplication has been a major evolutionary force shaping immunity related genes in dipterans [38]. Thus, the identification of putative IRGs in our dataset based solely on the inclusion of ortholog groups may be missing putative IRGs. Thus, by considering InterPro annotations, we found representative matches in 25 out of 27 families, representing the recognition, signaling, regulation and effector immune response processes.

**Figure 7:**
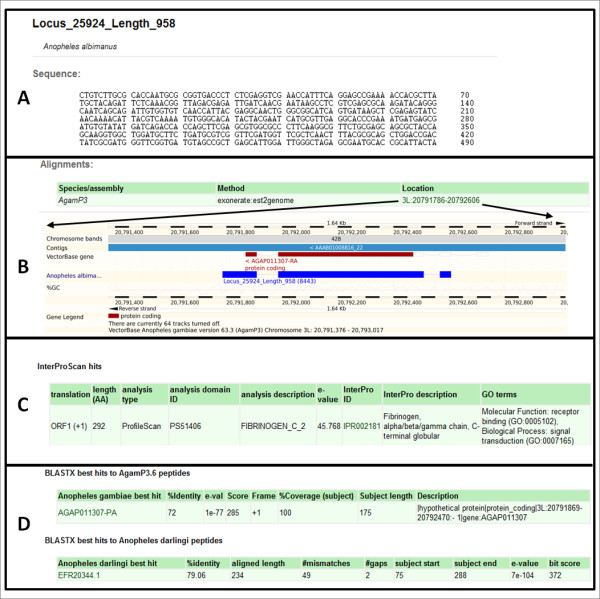
**Genome de-linked annotation viewer.** Screen shots of the *An. albimanus* transcript Locus_25924_Length_958 corresponding to the *FREP3* ortholog. **A)** Sequence pane. **B)** Alignment pane. An exonerate alignment to the *An. gambiae* reference genome can be displayed via the Distributed Annotation System (DAS) in the VectorBase genome browser, by following the link. The lower panes correspond to annotations which include InterPro annotation **(C)**, and BLASTX comparisons **(D)** to *An. gambiae*, *An. darlingi*, *D. melanogaster*, *Ae. aegypti*, *C. quinquefasciatus*, *I. scapularis* and *P. humanus*. For A, C and D only partial information is shown.

Among the ‘recognition phase’ genes, different pathogen recognition molecules were identified such as six Peptidoglycan Recognition Proteins (PGRPs), including the putative *PGRP-LD* ortholog [52]; three 1-3 β-glucan binding proteins (BGBPs), including the *GNBPB2* and *GNBPA1* orthologs [53]; six C-type lectins (CTLs), including the *CTL6* ortholog [54] and 19 Fibrinogen Related Proteins (FREPs), including the *An. gambiae FREP3* ortholog (Locus_25924_Length_958), but not *An. gambiae FREP9* ortholog which has been implicated in the anti-*Plasmodium* response [55] (Figure 7). We further identified six thioester containing proteins (TEPs) based on InterPro annotation (IPR009048) [56] that were missed by the orthology approach. Proteins containing Leucine-Rich Repeats (LRR) are highly abundant in metazoans and are involved in molecular recognition in a wide variety of biological processes. As mentioned in the previous section, we identified 38 transcripts containing LRR domains (IPR001611). However, mosquitoes possess a unique type of LRR-domain among proteins that are involved in immune responses [57]. Apart from the LRR, these proteins share structural features including a conserved pattern of cysteine residues and coiled-coil domains. We found one putative Leucine Rich Repeat immune protein (LRIM) that displays structural features of the Long LRIM subfamily and two that show compatible features with the short LRIM subfamily, including the *LRIM6* putative ortholog (Locus_11561_Length_1221) [57].

The major immune response signal transduction pathways in dipterans are the Toll and the Imd pathways. Gene members of these pathways tend to be better conserved in different mosquito species than genes implicated in the recognition or effector phases [38,58]. Although transcripts containing LRR or Toll/interleukin-1 receptor homology (TIR) domain (IPR000157) were found, a clear Toll receptor homolog was not. However, we found important members of the Toll pathway such as *PELLEMYD88* and *TRAF6*; the Imd pathway such as *CASPARIKK1* and *IKK2*[38] and STAT pathway such as *DOMESOCS* and *STAT2*[59,60] (Additional file 1), as well as *REL2* transcription factor [61].

Autophagy was originally described as a cellular response to starvation. However, it has recently been shown to be a critical process related to immune and stress responses, clearance of intracellular pathogens and damaged organelles, as well as cell survival. There are several genes involved in the induction of autophagy, autophagosome nucleation, autophagosome expansion and autophagosome recycling [62]. There are 20 *An. gambiae* entries in the ImmunoDB related to autophagy. Given the importance of all the mentioned processes in malaria transmission, we searched the ImmunoDB for orthologs and found 12. In the induction of the pathway, only the *TOR* homolog was found. Similarly, the only homolog involved in the nucleation phase that we found was the BCL-2 homolog *DEBCL* (*buffy* in *D. melanogaster*) [63]. The remaining ten corresponded to genes involved only in autophagosome expansion such as *APG3APG4AAPG4BAPG7AAPG7BAPG8* and *APG16* orthologs or autophagosome recycling such as *APG2APG9* and *APG18* (Additional file 1). In contrast, we found a relative depletion of caspases (only *CASPS7* and *CASPL1*, of 14 caspases in *An. gambiae*) and the absence of caspase activators (*AKR* and *Michelob_x*), suggesting that, as in larvae midgut [64], autophagy could be a more active homeostatic tissue process than apoptosis in adult mosquitoes.

## Conclusions

We have explored the adult female transcriptome of an important New World malaria vector, *An. albimanus*, by sequencing cDNA libraries generated from different tissue sources related to the *Plasmodium* life cycle such as midgut, cuticular fat body, dorsal vessel and salivary gland. Merging Sanger and NGS data into a single assembly generated a robust dataset with adequate transcript lengths that could be effectively mapped to the *An. darlingi* genome, covered 90% of the core eukaryotic genome and half of the predicted proteome of other mosquito vectors. We identified protein-coding transcripts involved in biological processes such as immune recognition, immune signaling pathways, insecticide resistance and autophagy that may be relevant to the *Plasmodium* cycle or may represent targets for novel control strategies. As a result of this work, the genomic information available for *An. albimanus* has increased several hundred-fold, thus providing molecular inputs for research in this species: 1) from a single gene perspective; 2) to gain insight into the anopheline radiations in the New World; 3) facilitating further genomic and proteomic approaches; and 4) assisting gene finding and validation of the *An. albimanus* genome in the context of the *Anopheles* cluster genome sequencing project [2]. Sequence information, predicted proteome comparisons, transcript mapping to the *An. gambiae* genome and InterPro annotations described in this manuscript are accessible to the community through the VectorBase website (http://www.vectorbase.org/Other/AdditionalOrganisms/).

## Materials and methods

### Mosquitoes and mosquito infections with *P. vivax*

All the mosquito samples used in the present work were 3-5 day post-emergence female *An. albimanus* of the white-stripe strain [65] obtained from the insectary of the National Institute of Public Health (INSP) in Cuernavaca, México. Mosquitoes were fed with 10% sucrose *ad libitum* and reared in a 12:12 h light cycle maintained at 28 °C and 80% relative humidity.

Mosquito infections with *P. vivax* CSP-VK210 and extraction of midgut epithelium of 24 h and seven days after an infectious blood meal were performed as described [66] in the insectary of the Centro Regional de Investigación en Salud Pública (CRISP) in Tapachula, Chiapas, México, according to Institute ethical guidelines and approval.

### cDNA libraries for Sanger sequencing

To capture transcripts from mosquito organs that are relevant for the *Plasmodium sp* life cycle, three cDNA libraries were generated for Sanger sequencing: A sucrose-fed female midgut (site of invasion) and a salivary gland (site of sporozoite maturation and inoculation to vertebrate host) cDNA libraries were constructed from 5 μg of total RNA. cDNA was ligated to Uni-ZAP XR vector (Stratagene). The phage library was mass excised and plated into LB-agar plates. Individual colonies were replicated in 384-well plates. A third cDNA library was constructed from 0.5 μg of Poly A + RNA extracted from whole female *An. albimanus* 12 h after inoculation with 0.0004 OD of *Serratia marcescens* in the hemocoel, which elicits an immune response that limits the development of *P. vivax*[67]. cDNA synthesis and library construction was done using the Creator SMART cDNA Library Construction Kit (Clontech) according to the manufacturer instructions. The library was transformed in *Escherichia coli* by electroporation and plated in LB-Agar with chloramphenicol (30 μg/mL). Individual colonies were replicated in 384-well plates.

### Template preparation and Sanger sequencing

*E. coli* clones were inoculated in CIRCLEGROW® (Krackler scientific) liquid media with either chloramphenicol (pDNA-lib) or ampicilin (pBluescript) at 37 °C in 96-well plates for 16 h. Plasmid DNA was prepared by alkaline lysis in Millipore filters and ethanol-precipitated and suspended in sterile deionized water. Sequencing was performed with fluorescent dye terminators in a 3100 Genetic Analyzer (Applied Biosystems).

### EST processing

Raw chromatogram files were quality assessed and trimmed with Phred using the trim_alt command with default parameters [68] and then converted to FASTA files using PH2FASTA. Vector sequence and linker sequences were removed using CrossMatch [69] and SeqClean [70]. Identification of mitochondrial and ribosomal protein transcripts was done by BLASTn searches to mosquito mitochondrial genomes or ribosomal protein sequence databases and filtered out. ESTs were submitted to dbEST at NCBI (Accession Numbers EV406110.1 - EV410194.1).

### 454 sequencing

Abdominal cuticles and the underlying fat body were obtained from 20 female adult mosquitoes and total RNA was extracted with TRIzol (Life Technologies). Integrity of RNA was verified in the Agilent Bioanalyzer standard RNA chip. One μg of RNA was used for full length RT-PCR amplification using the Super-SMART PCR cDNA synthesis kit (Clontech) according to the manufacturer’s instructions. The PCR amplified cDNA library was fragmented by nebulization and subjected to library preparation according to the 454 shotgun sequencing protocol. After emulsion PCR titration and amplification, the library was sequenced in a full picotiter plate using the Genome Sequencer FLX platform. A similar approach was used to generate additional midgut libraries from *P. vivax* infected-mosquitoes at 24 h post-infective blood meal (PIBM) and seven days PIBM, but sequenced as pooled bar-coded libraries in half picotiter plate. A third sequencing 454 run was performed with two cDNA libraries from dissected dorsal vessels, obtained at 18 h post-inoculation (intra-hemocoelic) with 0.25 μl of soluble fraction of zymosan (10 μg glucose-equivalents/ml, Sigma) as described [71] and saline-inoculated mosquitoes respectively. Dorsal vessels were collected in RNAlater (Ambion) and stored at -80°C. After RNAlater removal, total RNA was extracted with the RNAeasy Kit (Qiagen) and amplified with the SMARTER Pico PCR cDNA synthesis Kit (Clontech), and sequenced in a full picotiter plate (one region per library) using the GS FLX Titanium platform. Primer adaptors used for cDNA library generation were trimmed after signal processing using SeqClean. 454 sequence data was submitted to the Sequence Read Archive (SRA) (Accession number: SRA052091).

### Illumina sequencing

Total RNA from 50 *An. albimanus* midguts was extracted using TRIzol, DNased and cleaned with an RNAeasy column (Qiagen) according to the manufacturer’s instructions. Total RNA was then quality controlled for integrity on a Bioanalyzer (Agilent Technologies). mRNA libraries were constructed and sequenced, as previously described [72-74] on a single lane of a Illumina HiSeq 2000, which generated ~210 million 101 bp paired end reads. Illumina sequence data was submitted to the Sequence Read Archive (SRA) (Accession number: SRA051893).

### Assembly

The entire Illumina read set was split into eight equal sized read sets. Each one of these Illumina read sets was merged with the 454 and Sanger data and assembled using the Velvet [75] and Oases [76] software packages using three different kmer sizes (43, 45 and 47). The resulting contigs for each assembly were run again through Velvet and Oases to produce a final assembly. We then filtered the final assembly to retain only those loci that contained a single transcript, that were longer than 300 bp, and that had confidence scores of 1.0. To address which contigs contained 454 or Sanger reads, all 454 and Sanger reads were re-mapped to the initial assembly using the GS Reference Mapper v.2.5.3 using default parameters. Unmapped reads were re-assembled using the GS assembler v2.5.3 on cDNA mode to yield an additional set of 935 contigs.

### Genome mapping

The *An. gambiae* (AgamP3) [77] and *An. darlingi* genomes [18] were softmasked with RepeatMasker [78]. *An. albimanus* transcripts were aligned to either genome using Exonerate v. 2.2 [79] with the EST2 Genome mode, and a threshold score of 300, and maximum intron length of 20,000 bp.

### Transcript annotation

Gene ontology annotations were performed using Blast2Go [34]. For the Initial BLASTX against the NCBI-nr database the command-line option “-e1-e-6” was used. Additionally, transcripts were annotated according to the InterPro databases using InterProScan [35] in six-frame translation mode. Kruskal-Wallis test followed by Dunn’s correction was performed to calculate statistical differences within GO classes and protein percent identity with the Graph Pad PRISM software.

### *In silico* proteome comparison

The entire assembled transcript dataset was used to search for the best hit homologous proteins (BLASTX cut-off e-value 1.0E^-5^) in the *An. gambiae* (AgamP3.6), *Ae. aegypti* (AaegL1.2); *Culex quinquefasciatus* (CpipJ1.2), *P. humanus* (PhumU1.2) and *Ixodes scapularis* (IscaW1.1) predicted proteomes present at VectorBase [77], as well as the *An. darlingi*[18] and *D. melanogaster*[80] proteomes. Ortholog prediction was done by performing BLASTX and TBLASTN bidirectional comparisons between *An. albimanusAn. darlingi* and *An. gambiae* (e value 1.0E^-5^) to identify the best reciprocal hits within the three species.

To identify the proportion of the core eukaryotic genome covered by the *An. albimanus* transcriptome, we used HMM profiles corresponding to the 458 core eukaryotic proteins as provided by the CEGMA algorithm [32]. Local HMMER3 searches [81] were calibrated using the *An. gambiae* core eukaryotic protein validated dataset consisting of 453 sequences [82]. HMMER3 was performed using hmmscan command and the “-T 40” and “--domT 40” filters against the *An. albimanus* predicted proteome, as well as the predicted proteomes of *An. darlingiAn. gambiaeAe. aegyptiC. quinquefasciatus* and *D. melanogaster*.

### Web interface

Many web-based genome browsers [83-85] are available as open source software and are well suited to displaying transcript annotations. However they are heavily dependent on the availability of a genome sequence to act as a coordinate system. It is possible to adapt genome browsers to work without genomes [86] but it is not easy to keep them synchronized with developments in the “parent” software. In this work, we chose to develop a standalone “genome-free” web application, called GDAV (Genome-Delinked Annotation Viewer). It was designed with flexibility in mind; it can handle any kind of sequence annotations and integrates with genome browsers of closely related species via the DAS protocol [87]. The free open source code is available at https://github.com/VectorBase/GDAV and the software was developed within the auspices of VectorBase [77].

The key to GDAV’s flexibility is its use of three simple, open text file formats for loading data: FASTA for sequences, tab-delimited files for annotations and GFF3 for genome alignments. Only the loading of one or more FASTA files is mandatory, thereafter zero or more annotation and alignment files may be loaded into GDAV’s small MySQL schema using the supplied Perl scripts. The annotation file consists of rows of data identified by the sequence ID in the first column, and subsequent named columns providing arbitrary text annotations. The Java-based web interface is simple to deploy within a Java web server such as Apache Tomcat. The web interface, with its integrated search facility, treats all annotations as plain text—no special treatment of numeric data (e.g. range queries or unit conversions) is provided. Link-outs to third party databases from specific columns containing suitable IDs are possible through the configuration file. A Java-based DAS server based on Dazzle [88] is bundled with GDAV. It can be used to display GFF3 file-derived gapped alignments (e.g. exon-intron structure) of the sequences stored in GDAV with respect to the genomes of one or more closely related species. Any alignment features shown via DAS in genome browsers link back to the sequence report page in GDAV.

In this study, the annotation files loaded into the system include the InterPro domain assignments and the BLASTX results providing “best hits” to several other proteomes. The GFF3-format exonerate alignments described above were also loaded into the system.

## Abbreviations

BGBP: β-glucan Binding Proteins; CEG: Core Eukaryotic Gene; CTL’s: C-Type Lectin; DAS: Distributed Annotation System; FREP: Fibrinogen Related Protein; GDAV: Genome De-linked Annotation Viewer; GO: Gene Ontology; GST: Glutathion-S-Transferase; HSP: High Scoring Pair; IRG’s: Immune Response Genes; LRIM: Leucine Rich Repeat Immune Protein; LRR: Leucine Rich Repeat; My: Million Years; NGS: Next Generation Sequencing; PGRP: Peptidoglycan Recognition Protein; UTR: Untranslated Region.

## Competing interests

None declared.

## Author contributions

JMB conceived the study, participated in the generation of some cDNA libraries, performed data analysis, helped in designing web application specification and drafted the manuscript. MHR, RRD, AR and CAM contributed to study conception and design and helped drafting the manuscript. JGG performed transcriptome assembly. RMM performed InterPro annotation and in conjunction with SNR designed the web application specification and developed the web application, database and curation tools. CU, CAM and DEG participated in data analysis and interpretation. REG, JTS, MOM, LGC, SHM and FCR participated in mosquito experiments design, cDNA library generation, 454 and Sanger sequencing and data analysis. All authors read and approved the final manuscript.

## Supplementary Material

Additional file 1 Immunity related putative ortholog genes^a^. Click here for file
